# The Crown Prince William and His Left Arm

**Published:** 1888-07-21

**Authors:** 


### THE CROWN PRINCE WILLIAM AND HIS LEFT ARM.

ROYAL personages are public property, and "the fierce white light that beats upon a throne" takes note of physical defects as well as of intellectual and moral qualities. Recently the light has been shining on the Crown Prince of Germany, and sending a special ray to investigate the cause of his left arm being shorter than his right. There are two versions of the accident; the first, and popular one, is given by a correspondent of the New York *Medical Record*, and is to the effect that the Prince's arm was broken during his birth, which was a long and difficult one, either by the unsuccessful efforts of the physician-in-ordinary in attendance on the Crown Princess to effect version, or by Professor Edward Martin, who was at last called in, after too long a delay, to assist, and succeeded in the required operation. Dr. B. Scharlan, however, who knew Professor Martin well, contradicts this statement, and says Dr. Martin always assured him that the Prince was born with a congenital atrophy of the left arm. On subsequent occasions, however, the Crown Princess was always attended by an English doctor, chosen by Queen Victoria, and this gave rise to the rumour that Dr. Martin had broken Prince William's arm, and either overlooked the fact, or left it unmentioned, thinking that it would heal spontaneously, and that he had thereby lost the patronage of the Royal Family.

